# X-linked polymorphisms in *TLR7* and *TLR8* genes are associated with protection against Chikungunya fever

**DOI:** 10.1590/0074-02760230224

**Published:** 2025-06-13

**Authors:** Wilker Jose Perez Gotay, Mariella Sousa Coêlho Maciel, Raphael de Oliveira Rodrigues, Cynthia Chester Cardoso, Caroline Nobre Oliveira, Artur Fontenelle Lima Montenegro, Juliana Navarro Ueda Yaochite

**Affiliations:** 1 Universidade Federal do Ceará, Faculdade de Farmácia, Odontologia e Enfermagem, Departamento de Análises Clínicas e Toxicológicas, Fortaleza, CE, Brasil; 2 Universidade Federal do Rio de Janeiro, Instituto de Biologia, Laboratório de Virologia Molecular, Rio de Janeiro, RJ, Brasil

**Keywords:** Chikungunya, single nucleotide polymorphisms, toll-like receptors (TLRs), interferon regulatory factor 5 (IRF-5), immune response

## Abstract

**BACKGROUND:**

Chikungunya virus (CHIKV) causes an infection that leads to the activation of the innate immune response, triggering receptor pathways such as toll-like receptors (TLRs).

**OBJECTIVE:**

The present study aimed to investigate the association of single nucleotide polymorphisms (SNPs) in genes encoding toll-like receptors 3, 7, and 8 and *IRF5* in susceptibility to CHIKV infection and persistent joint pain.

**METHODS:**

A case-control study was carried out. The study included 121 symptomatic cases, 29 asymptomatic cases, and 182 healthy controls matched for age and sex. Polymorphisms were identified by TaqMan^®^ SNP Genotyping assays.

**FINDINGS:**

The G allele of the *TLR7* variant (rs3853839 G/C) and the G allele of *TLR8* (rs3764879 G/C) were associated with protection against CHIKV infection [_adjusted_ odd ratio (OR) = 0.64; p = 0.02 and _adjusted_ OR = 0.54; p = 0.001, respectively]. Moreover, individuals who presented the G allele in the rs3764879 variant have a greater chance of developing the asymptomatic form (_adjusted_ OR =2.88; p =0.004). The development of persistent joint pain was not associated with any investigated SNPs in positive anti-CHIKV IgG individuals.

**MAIN CONCLUSIONS:**

This study identified *TLR7* and *TLR8* gene polymorphisms as protective factors for Chikungunya infection.

The Chikungunya virus (CHIKV) is a member of the *Alphavirus* genus, belonging to the *Togaviridae* family, whose genetic material is constituted by a single-strand RNA of approximately 12 kb with positve polarity.^1^ ^,^ ^2^ The female mosquito, through the bite, introduces the virions at the intradermal level, and they enter the subcutaneous capillaries.^1^ Chikungunya fever is an acute febrile illness associated with severe and frequent debilitating polyarthralgia caused by the CHIKV infection.^3^ The most prominent symptoms of CHIKV infection are severe joint swelling and persistent joint pain lasting longer than three-six months after infection.^4^ ^,^ ^5^

The innate antiviral response against viruses primarily begins with the recognition of viral RNA by pattern recognition receptors (PRRs), as the nucleotide oligomerisation domain (NOD), retinoic acid-inducible gene I (RIG-I), and endosomal toll-like receptors (TLRs 3/7/8) that play a key role in the detection of these agents and subsequent induction of effector responses by antiviral molecules, as IFN-α and IFN-β.^6^ ^,^ ^7^ TLR-3 recognises double-stranded RNA, whereas TLR-7 and TLR-8 recognise single-stranded RNA.^8^ There is evidence that CHIKV infection activates TLR pathways in primary human monocytes and monocyte-derived macrophages, playing a crucial role in the pathogenesis of CHIKV.^9^ An experimental study showed an increase in the level of TLR-3 transcription during CHIKV infection resulting in the elimination of the virus in nine days.^10^ Stimulation of TLR-3 leads to the activation of the transcription factor NF-kB and the production of pro-inflammatory cytokines such as tumour necrosis factor (TNF), IL-6, and IL-12.^11^ ^,^ ^12^ After ligand binds to TLR7/8, MyD88 is recruited along with IRAK1/4, and TRAF6 leads to autophosphorylation of IRAK4 and ubiquitination of interferon regulatory factor 5 (IRF5). This action results in homodimerisation and translocation of the interferon regulatory factor 5 (IRF5) homodimer to the nucleus, leading to the production of cytokines such as TNF, IL-12, and type I interferon (IFN-I).^5^ ^,^ ^13^

Single-nucleotide polymorphisms (SNPs), depending on their location, can influence the function of TLRs. If the variation is found in the coding region of the ectodomain of the receptor, it can affect its binding to the ligand. In contrast, the presence of the SNP in the region coding for the transmembrane domain or in the cytoplasmic domain can alter the ability to transmit the signal to the interior of the cell and its interaction with intracellular adapter molecules.^14^

A comprehensive study (systematic review/meta-analysis) demonstrated the impact of genetic variants (*TLR*s, *HLA*, and other immunological-related genes) on the course of CHIKV infection. The authors conclude that the literature about CHIKV infection and genetic variations is scarce, and most of the studies were conducted in India.^15^

A study carried out by Bucardo and colleagues showed that the *TLR3* rs3775291 polymorphism can influence the risk of Chikungunya fever in Nicaragua population.^12^ Case-control evaluations showed that the SNP in *TLR7* rs3853839 and *TLR8* rs3764879 can influence the susceptibility to the development of Chikungunya in Calcutta population.^5^ A meta-analysis study showed that the SNP rs2004640, a variant of the *IRF5* gene, was associated with rheumatoid arthritis especially when the dominant genetic model was applied.^16^ To date, there are no published studies that investigated the influence of *TLR3/7/8* and *IRF5* genes SNPs during CHIKV infection in the Brazilian population.

In light of the foregoing, this study aimed to identify the role of *TLR3* rs3775291, *TLR7* rs3853839, *TLR8* rs3764879, and *IRF5* rs2004640 polymorphisms in the susceptibility of CHIKV infection and the association of these polymorphisms with persistent joint pain in a population from northeastern Brazil (Fortaleza city/Ceará State).

## SUBJECTS AND METHODS

*Study participants* - A case-control study included individuals (≥ 18 years, n = 121) with at least three clinical symptoms of Chikungunya, anti-CHIKV IgG positive serology, and those affected by the disease over the years 2016, 2017, or 2018. The group of patients with persistent joint pain (n = 86/121) were patients who presented pain ≥ 3 months.

The control group consisted of individuals (≥ 18 years, n = 182) who never had any characteristic signs or symptoms of Chikungunya and were also negative for anti-CHIKV IgG serology. The asymptomatic group (n = 29) was composed of individuals who did not report clinical symptoms but were positive for IgG anti-CHIKV. Subjects were recruited at the Laboratory of Clinical and Toxicological Analysis (LACT) of the Federal University of Ceará, and 10 mL of peripheral blood was collected. All study subjects belonged to the same geographic area (State of Ceará, Brazil). Individuals with other infections (hepatitis B, hepatitis C, among others), pregnant women, immunosuppressed individuals, or those with neoplasms were excluded.

The information on the symptoms suffered by the patients during the CHIKV infection was obtained through the reports offered during the questionnaire application before the blood was drawn. Information was collected on sex, age, place of residence, telephone number, city of residence, number of affected joints, and date of onset of symptoms, duration of symptoms, the intensity of pain, pre-existing diseases, whether serology was performed for the diagnosis, and the medications used.

The risk of CHIKV infection was evaluated in anti-CHIKV IgG positive individuals (n = 150) versus anti-CHIKV IgG negative individuals (n = 182).

The development of persistent joint pain was investigated in anti-CHIKV IgG positive individuals with persistent joint pain (n = 86) versus anti-CHIKV IgG positive individuals without persistent joint pain (n = 64).

For comparisons between asymptomatic (ASYM) and symptomatic (SYM) individuals, the SYM group was composed by anti-CHIKV IgG positive symptomatic individuals (n = 121) and anti-CHIKVIgG positive asymptomatic individuals (n = 29) represented the ASYM group.

*Declaration of ethics* - This study was conducted after approval from the Research Ethics Committee of the Federal University of Ceará (PROPESQ/UFC, protocol number 3.212.740).

*Anti-CHIKV virus IgG detection* - Chikungunya-specific IgG was determined in 100 µL of serum diluted into 1:100 using a commercial capture enzyme-linked immunosorbent assay (ELISA) kit distributed by Euroimmun (EI 293a‐9601 G; Euroimmun AG, Brazil). The procedure and cut-off levels were carried out according to the manufacturer’s instructions. The kit for detecting anti-CHIKV IgG (Euroimmunn EIA) used in the present study presents overall accuracy of 88.8%, with a sensitivity of 100% [_95%_ confidence interval (CI) = 73.2% - 100%] and specificity of 81.8% (_95%_ CI = 58.9% - 94%).^17^

Dengue and Zika serological profiles were not evaluated despite these diseases occurring in Brazil. However, these two diseases do not have chronic joint pain as a characteristic symptom.

*DNA extraction* - Genomic DNA was extracted from whole blood samples collected in EDTA tubes using the commercial kit Biopur Extraction Kit Mini Plus Spin - 250 (Biopur, Brazil) according to the manufacturer’s recommendations. DNA samples were stored at -20ºC until use.

*TaqMan* ^®^ *SNP genotyping experiment* - Polymorphisms in genes *TLR3* (rs3775291 C/T), *TLR7* (rs3853839 G/C), *TLR8* (rs3764879 G/C), *IRF5* (rs2004640 G/T), and *CTLA4* (rs5742909 C/T) were genotyped by real-time polymerase chain reaction (RT-PCR) using TaqMan™ pre-developed assays (Applied Biosystems^®^, California, EE. UU.) following the manufacturer’s instructions. RT-PCR was performed using a CFX96 Touch Real-Time PCR Detection System and Bio-Rad CFX Manager 2.1 software (Bio-Rad Laboratories AB). Cycling conditions were 95ºC for 5 min, followed by 40 cycles of 95ºC for 15 s, and 60ºC for 1 min. Ultrapure water was used as a negative control.

*Statistical analysis* - Statistical analyses were performed in the R software (version 3.4) with the “SNPassoc” package. The Hardy-Weinberg equilibrium (HWE) test of all SNPs was performed using online calculator (https://www.genecalculators.net/pq-chwe-genotypes.html). For SNPs located on autosomes, HWE analyses were performed with a control group. While for the SNPs located on the X chromosome, the HWE analyses were only performed with women in the control group. Clinical characteristics of infected individuals were estimated by Fisher’s exact test using 2×2 contingency. The association between each SNP and the outcomes of interest (CHIKV infection, asymptomatic disease, and persistent joint pain) was assessed by Logistic regression models using R software, version 4.2.1. Results were represented as Odds ratio (OR) along with a 95% CI (_95%_ CI).The results of logistic regression models (Adjusted OR) were adjusted for age and sex. P < 0.05 was considered statistically significant. Pairwise linkage disequilibrium (LD) patterns were determined using the r^2^ statistic.

The power estimates were performed using epiR package. Considering the sample size available and a minimum allele frequency of 0.15 among controls, which is suitable for the SNPs under analysis, the minimum OR values to achieve 80% of power were: 2.22 for comparisons between all patients and anti-CHIKV IgG negative controls; and 3.18 for comparisons between anti-CHIKV IgG positive patients with persistent joint pain versus anti-CHIKV IgG positive individuals without persistent joint pain. For comparisons between ASYM and (SYM individuals, considering the sample size available, the minimum OR would be 3.69 with a minor allele frequency of 0.25.

## RESULTS

*Sociodemographic and clinical characteristics of cases and controls* - According to the study design, the median age (interquartile range) was 51 (27-63) years for controls, 57 (48-68) for SYM cases, and 58 (40-70.5) for ASYM cases (Table I). Concerning pre-existing diseases, there was an unequal distribution of these comorbidities among the different groups. The group of Chikungunya SYM individuals presented a higher frequency of chronic diseases, such as hypertension (45%), diabetes (33%), asthma (2%), anaemia (3%), cardiomyopathy (8%), and hypothyroidism (9%) compared to healthy controls (data not shown). Both SYM cases and health controls reported the presence of household contacts with CHIKV infection, as shown in Table I.

**TABLE I:** General characteristics of study participants

Characteristic	Study subjects, N**º**. (%)				p-value^*b*^
SYM (n = 121)	ASYM cases (n = 29)	Healthy controls (n = 182)	p-value^*a*^	p-value^*c*^
Sex						
Male	35 (29)	15 (51.7)	51 (28)	0.027^*^	0.89	0.016^*^
Female	86 (71)	14 (48.3)	131 (72)			
Age years (median and IQR)	57 (48 - 68)	58 (40 - 70.5)	51 (27 - 63)	0.82	0.0002^*^	0.12
Chronic diseases						
Yes	75 (62)	20 (69)	30 (16)	0.52	p < 0.0001^*^	p < 0.0001^*^
No	46 (38)	09 (31)	152 (84)			
Household contacts with CHIKV						
Yes	90 (74)	8 (27.6)	124 (68)	p < 0.0001^*^	0.25	p < 0.0001^*^
No	31 (26)	21 (72.4)	58 (32)			

Characteristic analyses were performed using Fisher’s exact test and Mann-Whitney test for age analysis, ^*^p < 0.05. p-value^*a*^: SYM cases versus ASYM cases; p-value^*b*^: SYM cases versus health controls; p-value^*c*^: ASYM cases versus healthy controls. Data represent No. (%) of study subjects, unless otherwise specified; p-values, IQR: interquartile range and chronic diseases including hypertension, diabetes, asthma, or arthritis; SYM: symptomatic; ASYM: asymptomatic; CHIKV: Chikungunya virus.

The prevalence of reported symptoms by SYM case group was fever (85%, 103/121), headache (72%, 87/121), and rash (42%, 51/121). All patients (100%, 121) reported joint pain during the disease. Pain intensity was classified as mild in 7% (9/121) of the patients, moderate in 23% (28/121), and severe in 69% (84/121). A total of 71% (86/121) of patients reported having suffered persistent joint pain. The most affected joints were the neck, shoulder, elbow, hand, fingers, knee, and ankle. A total of 55% (67/121) of cases reported joint oedema during Chikungunya manifestations, and 66% (80/121) of cases reported having suffered from diarrhoea during the course of the disease (p = 0.01), as shown in Table II.

**TABLE II:** Clinical characteristics of symptomatic (SYM) individuals

Frequency of symptoms					
Symptoms	SYM cases (n = 121)	Female (%) (n = 86)	Male (%) (n = 35)	Prevalence of Chikungunya symptoms (%)	p-value
Fever	103	75 (87)	28 (80)	103 (85)	0.40
Headache	87	63 (73)	24 (69)	87 (72)	0.66
Myalgia	81	61 (71)	20 (57)	81(67)	0.20
Rash	51	37 (43)	14 (40)	51 (42)	0.84
Joint pain	120	85 (99)	35 (100)	120 (99)	1.00
Persistent joint pain	86	63 (73)	21 (60)	86 (71)	0.19
Retro-ocular pain	53	41 (48)	12 (34)	53 (44)	0.23
Oedema	67	51 (59)	16 (48)	67 (55)	0.23
Vomiting/ Nausea	48	35 (41)	13 (37)	48 (55)	0.84
Weakness	75	55 (64)	20 (57)	75 (62)	0.54
Diarrhoea	80	50 (93)	30 (86)	80 (66)	0.01^*^

Clinical characteristic analyses were performed using Fisher’s exact test, ^*^p < 0.05.

Of the 121 cases, 86 were women, of whom 64 (74%) presented persistent joint pain; 35 were men, of whom 22 (63%) presented persistent joint pain. Concerning the control group of the 182 individuals, 131 were women (72%) and 51 were men (28%). Of the 29 individuals in the asymptomatic group, 14 were women (48%) and 15 were men (52%). These results are shown in Supplementary data (Figure).

*Association of TLR7 rs3853839 and TLR8 rs3764879 polymorphisms as protective factors against Chikungunya infection* - The results of Hardy-Weinberg equilibrium analysis are shown in Supplementary data (Table I). Linkage disequilibrium analyses were performed for the SNPs rs3764879 (*TLR8*) and rs3853839 (*TLR7*), and evidence of strong linkage disequilibrium between these markers was observed (D’ = 0.99 and r2 = 0.98). These results are shown in Supplementary data (Table II).

The statistical analyses allowed establishing the genotypic frequencies of the *TLR3* and *IFR5* polymorphisms. The results of association obtained through several models for these variants did not show statistically significant differences. No deviations from Hardy-Weinberg equilibrium were observed in the control group, and genotype frequencies are shown in Table III.

**TABLE III:** Genotypic association of the TLR3/7/8 and IRF5 single nucleotide polymorphisms (SNPs) with the risk of Chikungunya virus (CHIKV) infection

SNP	Genotype	SYM + ASYM cases (%) (n = 150)	Healthy controls (%) (n = 182)	Adjusted OR (95% CI)	p-value
*TLR3* (L412F rs3775291;1234 C > T)	All				
	Codominant				
	C/C	65 (43.3)	87 (47.8)	1.0 (Reference)	
	C/T	67 (44.7)	80 (44.0)	1.12 (0.71 - 1.77)	0.465
	T/T	18 (12.0)	15 (8.2)	1.61 (0.75 - 3.42)	
	Dominant				
	C/C	65 (43.3)	87 (47.8)	1.0 (Reference)	
	C/T-T/T	85 (56.7)	95 (52.2)	1.20 (0.78 - 1.85)	0.415
	log-Additive (T)	150 (45.2)	182 (54.8)	1.23 (0.88 - 1.73)	0.23
*IRF5* (198 G > T rs2004640)	All				
	Codominant				
	G/G	46 (30.7)	49 (26.9)	1.0 (Reference)	
	G/T	73 (48.7)	89 (48.9)	0.87 (0.53 - 1.45)	0.652
	T/T	31 (20.7)	44 (24.2)	0.75 (0.41 - 1.38)	
	Dominant				
	G/G	46 (30.7)	49 (26.9)	1.0 (Reference)	
	G/T-T/T	104 (69.3)	133 (73.1)	0.83 (0.52 - 1.34)	0.453
	log-Additive (G)	150 (45.2)	182 (54.8)	1.22 (0.89 - 1.68)	0.20
*TLR7* (3′UTR G > C rs3853839)	All	(n = 150)	(n = 182)		
	log-Additive (G)	150 (45.2)	182 (54.8)	0.64 (0.44 - 0.93)	0.02^*^
	FEMALE	(n = 100)	(n = 131)		
	Codominant				
	C/C	53 (53.0)	50 (38.2)	1.0 (Reference)	
	G/C	36 (36.0)	64 (48.9)	0.53 (0.30 - 0.93)	0.075
	G/G	11 (11.0)	17 (13.0)	0.61 (0.26 - 1.43)	
	Dominant				
	C/C	53 (53.0)	50 (38.2)	1.0 (Reference)	
	G/C-G/G	47 (47.0)	81 (61.8)	0.55 (0.32 - 0.93)	0.024^*^
	log-Additive (G)	100 (43.3)	131 (56.7)	0.69 (0.47 - 1.02)	0.060
*TLR8* (129 C > G rs3764879)	All	(n = 150)	(n = 182)		
	log-Additive (G)	150 (45.2)	182 (54.8)	0.54(0.38 - 0.78)	0.00^1^*
	FEMALE	(n = 100)	(n = 131)		
	Codominant				
	C/C	39 (39.0)	28 (21.4)	1.0 (Reference)	
	G/C	46 (46.0)	66 (50.4)	0.50 (0.27 - 0.92)	0.004^*^
	G/G	15 (15.0)	37 (28.2)	0.29 (0.13 - 0.63)	
	Dominant				
	C/C	39 (39.0)	28 (21.4)	1.0 (Reference)	
	G/C-G/G	61 (61.0)	103 (78.6)	0.43 (0.24 - 0.76)	0.003^*^
	log-Additive (G)	100 (43.3)	131 (56.7)	0.54 (0.36 - 0.78)	0.001^*^

The statistical analyses were performed using logistic regression models adjusted for age (continuous) and sex. Significant associations are highlighted in bold (^*^p < 0.05 at 95% CI was considered statistically significant). SYM: symptomatic; ASYM: asymptomatic; OR: odds ratio; CI: confidence interval.

Since *TLR7* and *TLR8* genes are located at the X chromosome, statistical analyses were first performed under an additive model to allow inclusion of male participants. These analyses showed a protective effect against Chikungunya infection for allele G at rs3853839 (_Adjusted_ OR = 0.64; _95%_ CI = 0.44-0.93; p = 0.02) and for allele G at rs3764879 (_Adjusted_ OR = 0.54; _95%_ CI = 0.38-0.78; p = 0.001) after adjusted for age and sex.

Afterward, data analysis was conducted, including only female subjects, and then different models were evaluated. Similarly, it shows that the G allele of the TLR7 polymorphism (rs3853839 G/C) and the G allele of the TLR8 polymorphism (rs3764879 G/C) confer protection against Chikungunya infection. The G allele of the SNP *TRL7* rs3853839 was also associated with a lower risk of CHIKV infection under the dominant model (_Adjusted_ OR = 0.55; _95%_ CI = 0.32 - 0.93; p = 0.024). The same trend was observed for *TLR8* rs3764879 variant, which showed a protective effect against Chikungunya infection for genotypes carrying allele G under codominant (_Adjusted_ OR = 0.50 and 0.29 for GC and GG genotypes, respectively; p = 0.004), dominant (_Adjusted_ OR = 0.43; _95%_CI = 0.24 - 0.76; p = 0.003), and log-additive (_Adjusted_ OR = 0.54; _95%_ CI = 0.36 - 0.78; p = 0.001) models. These results are shown in Table III.

*No association of TLR3 rs3775291, IRF5 rs2004640, TLR7 rs3853839 and TLR8 rs3764879 polymorphisms as a protective factor against persistent joint pain* - The development of persistent joint pain was investigated by comparing the individuals with this symptom and positive anti-CHIKV IgG serology (n = 86) versus individuals without this symptom and with anti-CHIKV IgG positive serology (n = 64). Using the additive model, the following results were observed for the G allele of *rs3853839* (_Adjusted_ OR = 0.72; _95%_ CI 0.42-1.26; p = 0.25); *rs3764879* (_Adjusted_ OR = 0.59; _95%_ CI 0.34-1.01; p = 0.06); and *rs2004640* (_Adjusted_ OR = 0.88; _95%_ CI 0.55-1.41; p = 0.60). Looking at *TLR3 rs3775291* SNP, the results for T allele were: _Adjusted_ OR = 1.08; _95%_ CI 0.66-1.77; p = 0.76. Thus, the investigated SNPs were not related to persistent joint pain development in infected individuals. These results are shown in Table IV.

**TABLE IV:** Genotypic association of TLR3/7/8 and IRF5 single nucleotide polymorphisms (SNPs) in positive anti-Chikungunya virus (anti-CHIKV) IgG individuals with persistent joint pain *versus* positive anti-CHIKV IgG individuals without persistent joint pain

SNP	Genotype	Positive anti-CHIKV IgG individuals without persistent joint pain (%) (n = 64)	Positive anti-CHIKV IgG individuals with persistent joint pain (%) (n = 86)	Adjusted OR (95% CI)	p-value
*TLR3* (L412F rs3775291;1234 C > T)	All				
	Codominant				
	C/C	29 (45.3)	36 (41.9)	1.0 (Reference)	
	C/T	28 (43.8)	39 (45.3)	1.12 (0.56 - 2.23)	0.892
	T/T	07 (10.9)	11 (12.8)	1.27 (0.44 - 3.68)	
	Dominant				
	C/C	29 (45.3)	36 (41.9)	1.0 (Reference)	
	C/T-T/T	35 (54.7)	50 (58.1)	1.15 (0.60 - 2.21)	0.673
	log-Additive (T)	64 (42.7)	86 (57.3)	1.08 (0.66 - 1.77)	0.760
*IRF5* (198 G > T rs2004640)	All				
	Codominant				
	G/G	19 (29.7)	27 (31.4)	1.0 (Reference)	
	G/T	35 (54.7)	38 (44.2)	0.76 (0.36 - 1.61)	0.320
	T/T	10 (15.6)	21 (24.4)	1.48 (0.57 - 3.84)	
	Dominant				
	G/G	19 (29.7)	27 (31.4)	1.0 (Reference)	
	G/T-T/T	45 (70.3)	59 (68.6)	0.92 (0.46 - 1.86)	0.822
	log-Additive (G)	64 (42.7)	86 (57.3)	1.15 (0.73 - 1.82)	0.544
*TLR7* (3′UTR G > C rs3853839)	All	(n = 64)	(n = 86)		
	log-Additive (G)	64 (42.7)	86 (57.3)	0.72 (0.42 - 1.26)	0.250
	FEMALE	(n = 36)	(n = 64)		
	Codominant				
	C/C	18 (50.0)	35 (54.7)	1.0 (Reference)	
	G/C	14 (38.9)	22 (34.4)	0.81 (0.34 - 1.95)	0.893
	G/G	4 (11.1)	7 (10.9)	0.90 (0.23 - 1.88)	
	Dominant				
	C/C	18 (50.0)	35 (54.7)	1.0 (Reference)	
	G/C-G/G	18 (50.0)	29 (45.3)	0.83 (0.37 - 1.88)	0.652
	log-Additive (G)	36 (36.0)	64 (64.0)	0.90 (0.50 - 1.64)	0.732
*TLR8* (129 C > G rs3764879)	All	(n = 64)	(n = 86)		
	log-Additive (G)	64 (42.7)	86 (57.3)	0.59 (0.34 - 1.01)	0.06
	FEMALE	(n = 36)	(n = 64)		
	Codominant				
	C/C	12 (33.3)	27 (42.2)	1.0 (Reference)	
	G/C	18 (50.0)	28 (43.8)	0.69 (0.28 - 1.70)	0.680
	G/G	06 (16.7)	09 (14.1)	0.67 (0.19 - 2.30)	
	Dominant				
	C/C	12 (33.3)	27 (42.2)	1.0 (Reference)	
	G/C-G/G	24(66.7)	37 (57.8)	0.69 (0.29 - 1.61)	0.381
	log-Additive (G)	36 (36.0)	64 (64.0)	0.79 (0.44 - 1.42)	0.429

Statistical analyses were performed using logistic regression models adjusted for age (continuous) and sex (^*^p < 0.05 to 95% CI was considered statistically significant). OR: odds ratio; CI: confidence interval.

*The rs3764879 polymorphism in the TLR8 gene is associated with ASYM CHIKV infection* - Of the 211 individuals who did not report Chikungunya symptoms, 182 presented negative serology for CHIKV and 29 (14%) exhibited seropositive samples for anti-CHIKV IgG (ASYM cases). Fourteen women (6.8%) and 15 men (7.2%) were considered ASYM. IgG for CHIKV was determined as a marker of infection and the stratification served to explore whether polymorphisms in different genes are associated with both SYM and ASYM infections.

Using the additive model, the G allele of rs3764879 in *TLR8* (129 C > G) was associated with ASYM disease (_Adjusted_ OR = 2.88; _95%_ CI 1.40-5.94; p = 0.004). Moreover, using the dominant model for female individuals, the G allele carriers were associated with ASYM infection (_Adjusted_ OR = 4.53; _95%_ CI = 0.96-21.49; p = 0.029). These results are shown in Table V.

**TABLE V:** Comparison of asymptomatic (ASYM) infection *versus* symptomatic (SYM) infection

SNP	Genotype	ASYM cases (%) (n = 29)	SYM cases (%) (n = 121)	Adjusted OR (95% CI)	p-value
*TLR3* (L412F rs3775291;1234 C > T)	All				
	Codominant				
	C/C	11 (37.9)	54 (44.6)	1.0 (Reference)	
	C/T	15 (51.7)	52 (43.0)	1.42 (0.60 - 3.37)	0.697
	T/T	3 (10.3)	15 (12.4)	0.98 (0.24 3.98)	
	Dominant				
	C/C	11 (37.9)	54 (44.6)	1.0 (Reference)	0.511
	C/T-T/T	18 (62.1)	67 (55.4)	1.32 (0.57 - 3.03)	
	log-Additive (T)	29 (19.3)	121 (80.7)	1.17 (0.63 - 2.17)	0.62
*IRF5* (198 G > T rs2004640)	All				
	Codominant				
	G/G	8 (27.6)	38 (31.4)	1.0 (Reference)	
	G/T	16 (55.2)	57 (47.1)	1.33 (0.52 - 3.42)	0.729
	T/T	5 (17.2)	26 (21.5)	0.91(0.27 - 3.11)	
	Dominant				
	G/G	8 (27.6)	38 (31.4)	1.0 (Reference)	0.686
	G/T-T/T	21 (72.4)	83 (68.6)	1.20 (0.49 - 2.96)	
	log-Additive (G)	29 (19.3)	121 (80.7)	0.99 (0.55 - 1.78)	0.976
*TLR7* (3′UTR G > C rs3853839)	All	(n = 29)	(n = 121)		
	log-Additive (G)	29 (19.3)	121 (80.7)	1.52 (0.76 - 3.06)	0.24
	FEMALE	(n = 14)	(n = 86)		
	Codominant				
	C/C	6 (42.9)	47 (54.7)	1.0 (Reference)	
	G/C	7 (50.0)	29 (33.7)	1.89 (0.58 - 6.18)	0.499
	G/G	1 (7.1)	10 (11.6)	0.78 (0.08 - 7.24)	
	Dominant				
	C/C	6 (42.9)	47 (54.7)	1.0 (Reference)	
	G/C-G/G	8 (57.1)	39 (45.3)	1.61 (0.51 - 5.03)	0.412
	log-Additive (G)	14 (14.0)	86 (86.0)	1.17 (0.52 - 2.62)	0.712
*TLR8* (129 C > G rs3764879)	All	(n = 29)	(n = 121)		
	log-Additive (G)	29 (19.3)	121 (80.7)	2.88 (1.40 - 5.94)	0.00^4^*
	FEMALE	(n = 14)	(n = 86)		
	Codominant				
	C/C	2 (14.3)	37 (43.0)	1.0 (Reference)	
	G/C	10 (71.4)	36 (41.9)	5.14 (1.05 - 25.10)	0.071
	G/G	2 (14.3)	13 (15.1)	2.85 (0.36 - 22.31)	
	Dominant				
	C/C	2 (14.3)	37 (43.0)	1.0 (Reference)	
	G/C-G/G	12(85.7)	49 (57.0)	4.53 (0.96 - 21.49)	0.029^*^
	log-Additive (G)	14 (14.0)	86 (86.0)	1.76 (0.79 - 3.94)	0.167

Statistical analyses were performed using logistic regression models adjusted for age (continuous) and sex (^*^p < 0.05 to 95% CI was considered statistically significant). SNP: single nucleotide polymorphism; OR: odds ratio; CI: confidence interval.

## DISCUSSION

The immunopathogenesis of CHIKV infection is not fully established yet. Host factors play an essential role in the progression and severity of the disease. Variants in genes related to the immune system, such as those involved in inflammatory and antiviral responses, can modify the efficiency of the body’s defence against the CHIKV. A review article was recently published by our research group, and it is possible to note that our genetic targets were studied by other groups and their significant influence on CHIKV infection in different populations.^18^ Furthermore, factors such as age and comorbidities may influence the disease severity.^19^ ^,^ ^20^ ^,^ ^21^

In the present study, the case and control groups were matched by age and sex. The median age (interquartile range) was 51 (27 - 63) years for the control group and 57 (48 - 68) years for the case group. Female individuals constituted 72% of the participants in the control group and 71% of the case group. These findings are similar to those shown by Bucardo et al., in which 64% of the control group subjects were female and also 66% of the patient cases.^12^ Regarding home contacts, 74% of the cases and 68% of the healthy controls reported having household contacts with CHIKV infection. This indicates that the population under study effectively lives in an area where CHIKV infection is endemic, meaning that all cases and controls have been exposed to CHIKV. Since case and control individuals are equally exposed to the virus, intrinsic factors may determine an individual’s susceptibility to the infection. Thus, SNPs may influence the risk of CHIKV infection.

Looking at pre-existing diseases, our analysis showed an uneven distribution of these comorbidities among the different groups (p < 0.001). A higher frequency of hypertension and diabetes was presented by symptomatic CHIKV-infected individuals. These results agree with those shown in the study carried out by Bucardo and colleagues, who showed that these inequalities of comorbidities among the groups could be a risk factor for CHIKV infection.^12^

Some studies have established a link between CHIKV infection and rheumatoid arthritis. It has been shown that around 1% of patients with chronic Chikungunya may develop rheumatoid arthritis.^16^ It has already been demonstrated that the genetic variant rs2004640, which is located in exon 1 of the *IRF5* gene, results in functional modifications that affect the expression of messenger RNA, increasing the risk of developing rheumatoid arthritis.^16^ In the current investigation, however, no differences were observed in the frequencies of alleles or genotypes of the rs2004640 polymorphism of the *IRF5* gene, as well as no association with the risk of CHIKV infection or persistent joint pain. To date, there are no reports in the literature about the influence of the SNP rs2004640 on arboviruses, only on infections caused by human immunodeficiency virus (HIV) and Epstein-Barr virus.^22^ ^,^ ^23^

The ability of humans to respond to pathogen-associated molecular patterns can be altered by various SNPs within the *TLR* genes of innate immunity, which could influence their functions, affecting the ability of microbial recognition and TLR signalling pathway.^24^ ^,^ ^25^

TLR-3 plays an important role in the immune response to infections caused by viruses. Studies have shown that TLR-3 regulates the host immune response and that loss of TLR-3 worsens disease in CHIKV infection.^26^ In the present study, it was not possible to observe differences in the frequencies of alleles or genotypes of *TLR3* gene polymorphism (rs3775291) and there was also no association with susceptibility to infection, persistent joint pain, or asymptomatic CHIKV infection. Silva and colleagues published a meta-analysis study to determine whether there is an association between the rs3775291 SNP in *TLR3* gene and susceptibility to infections. It is possible to note that the Asian and American continents showed that this SNP confers a higher risk against infections in a dominant genotypic model.^27^ This SNP was investigated during Zika infection,^28^ ^,^ ^29^ coronavirus disease 19 (COVID-19),^30^ hepatitis B and hepatitis C virus infections^31^ in the Brazilian population. However, there is no study conducted in the Brazilian population investigating the role of this SNP in Chikungunya settings.

Linkage disequilibrium analyses showed that the investigated variations at *TLR7* and *TLR8* genes are in almost perfect linkage disequilibrium (r^2^ = 0.98), suggesting that both variables segregate together indicating a high probability of joint inheritance. However it is not possible to predict which one has the main biological effect. There are some variations in the expression patterns of TLR-7 and TLR-8 receptors. TLR-7 is primarily expressed by plasmacytoid dendritic cells (pDCs), however, it is also present in myeloid and B cells. Myeloid cells strongly express TLR-8, but pDCs and B cells do not. This demonstrates that depending on which cell is being stimulated; TLR-7 and TLR-8 can drive distinct immune responses in response to microbes.

Some studies have demonstrated the role of rs3853839 (C/G) and rs3764879 (C/G) polymorphisms of *TLR7/8* genes in Chikungunya susceptibility and disease severity, respectively.^5^ ^,^ ^32^ In this context, our study represents the first report of *TLR7* and *TLR8* genetic variants associated with protection against CHIKV infection in the Brazilian population.

The findings of this investigation suggest that the G allele of *TLR7* rs3853839 and *TLR8* rs3764879 variants confer protection against CHIKV infection. The allelic frequency of the G allele for the *TLR7* genetic variant in the case group, for female individuals, was 47.0%, while for the control group it presented a frequency of 61.8% (p = 0,024). In the case group, the G allele for the *TLR8* variant showed an allelic frequency of 61.0% for female participants, but in the control group, the frequency was 78.6% (p = 0,003). Thus, the genotypes that carry the G allele (GC and GG) could present a protective effect or a reduced susceptibility to CHIKV infection. The research carried out by Shen and colleagues showed that the *TLR7* SNP rs3853839 C/G, located in the untranslated region 3ʼ(UTR), is associated with an increase in *TLR7* mRNA and, therefore, a greater expression of the TLR-7 protein. This study showed that T*LR7* transcripts are amplified to a greater extent in individuals carrying the G allele.^33^ Moreover, Yue and colleagues showed that the functional polymorphism of *TLR7* rs3853839 C allele was sex-specific and associated with protection against hepatitis C virus persistence among Chinese females, which may be due to specific IFN-α and IL-6 secretion profiles.^34^ The TLR8 -129C > G rs3764879 variant was examined in individuals with hepatitis C virus infection by Wang and colleagues. Greater TLR-8 protein expression and promoter activity were seen in those with the G allele, indicating that this SNP may be able to influence transcriptional regulation in monocytes. That explains why the natural course of the infection is inhibited in these hosts.^35^ The TLR-7/TLR-8 protein expression and activation pathways were not assessed in the present investigation. A possible explanation is that the G allele of *TLR7* rs3853839 and *TLR8* rs3764879 variants enhances TLR expression, which in turn improves CHIKV detection and the antiviral immune response in the control group.

In the current investigation, joint pain was reported by 99% of patients with CHIKV infection symptoms, and 71% of these patients had persistent joint pain. Dutta and Tripathi observed that 85.5% of CHIKV-infected patients exhibited joint pain and 80.3% had pain in seven joints. The SNPs analysis showed an association between the CT genotype of rs3775290 (*TLR3*) and the GG genotype of rs5741880 (*TLR7*) with joint pain.^5^ According to Bucardo and colleagues, older age, female sex and comorbid conditions were associated with risk of persistent joint pain. The rs1800629 variant in TNF-α gene acts as a risk factor for persistent joint pain. No association was observed between *CD209* (rs4804803) and *TLR3* (rs3775291) polymorphism and persistent joint pain.^12^ In the present study, *TLR3* rs3775291, *IRF5* rs2004640, *TLR7* rs3853839, and *TLR8* rs3764879 polymorphisms were not associated with protection against persistent joint pain in those who tested positive for anti-CHIKV IgG.

Our study showed a subclinical infection rate of 14% for women and 15% for men who reported no symptoms (ASYM) and who were Chikungunya IgG seropositive. According to a cross-sectional study one year after the introduction of Chikungunya in Central America, 19% of subclinical infections were reported.^12^ An interesting finding was that when comparing the asymptomatic individuals with the symptomatic group, female individuals carrying the G allele of *TLR8* rs3764879 variant presented a protective effect or reduced susceptibility to CHIKV infection.

Genomic studies carried out in different populations have demonstrated significant associations between the occurrence of SNPs in genes related to immune system molecules and the clinical development of Chikungunya. Ferreira and colleagues performed a meta-analysis to study the influence of genetic variations in genes related to immune response on infection outcomes of patients with CHIKV infection. The authors found a significant association between the *HLA DRB1* ^*^ *14* allele and susceptibility to SYM CHIKV infection in the Indian population compared to healthy controls.^15^ In that respect, it is worth highlighting that the distribution of genomic variants in the Brazilian population presents wide heterogeneity due to strong miscegenation.^18^ Thus, research with SNPs in different Brazilian states *makes* it possible to identify specific regional associations between genetic variants and disease outcomes, contributing to the development of more effective prevention, diagnosis, and treatment strategies tailored to each region’s needs. In Brazil, this is the first study to evaluate the influence of the rs3853839 and rs3764879 variants in the *TLR7* and *TLR8* genes, respectively, in Chikungunya setting.

The present study has some limitations. The main limitation of this study could be that the clinical data collection was based on the recall responses of the patients. This would not affect the analysis of SNPs and other risk factors of Chikungunya disease because its diagnosis was confirmed by laboratory methods. The ASYM group was limited, and a larger number of individuals are needed to better understand our results.

In conclusion, the present study indicated a lower risk of Chikungunya infection among individuals with certain genetic variants in the *TLR7* and *TLR8* genes. Moreover, female individuals carrying the G allele of rs3764879 in *TLR8* gene was associated with ASYM infection.
