# Supplementary material for: X-linked polymorphisms in TLR7 and TLR8 genes are associated with protection against Chikungunya fever
Source: Mem Inst Oswaldo Cruz. 2025 Jun 13;120:e230224. doi: 10.1590/0074-02760230224 (PMC12165714; doi:10.1590/0074-02760230224)
Supplement: Supplementary file 1 [file 1678-8060-mioc-120-e230224-s.pdf]

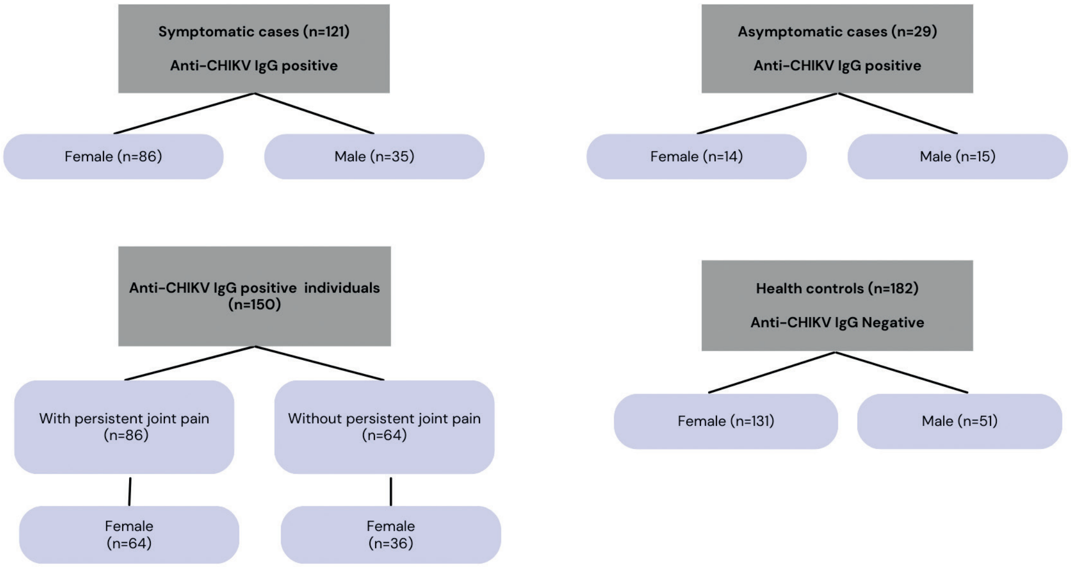

Schematic characterisation of study subjects.

TABLE I

Hardy-Weinberg equilibrium for the studied single nucleotide polymorphisms (SNPs) in healthy control samples

SNPs located on the autosomes			
SNP	Genotypes	N° (%) (n = 182)	Hardy-Weinberg Equilibrium (HWE) (p-value*)*
TLR3 (rs3775291 C/T)	CC<br>CT<br>TT	87 (47,80)<br>80 (43,96)<br>15 (8,24)	p = 0.569
IRF5 (rs2004640 G/T)	GG<br>GT<br>TT	49 (26,92)<br>89 (48,90)<br>44 (24,18)	p = 0.774
SNPs located on the X chromosome			
SNP	Genotypes	N° (%) (n = 131)	Hardy-Weinberg Equilibrium (p-value*)*
TLR7 (rs3853839 C/G)	CC<br>CG<br>GG	50 (38,17)<br>64 (48,85)<br>17 (12,98)	p = 0.620
TLR8 (rs3764879 G/C)	GG<br>GC<br>CC	37 (28,25)<br>66 (50,38)<br>28 (21,37)	p = 0.887

TABLE II

Linkage disequilibrium analyses for the single nucleotide polymorphisms (SNP) rs3764879 (*TLR8*) and rs3853839 (*TLR7*)

LD rs3764879 x rs3853839	
D	0.2487394
D’	0.9996761
Corr	0.9996761
X^2	261.8303098
p-value	< 2.2204e-16

CC BY
